# The positive reinforcing effects of cocaine and opposite-sex social contact: roles of biological sex and estrus

**DOI:** 10.1007/s00213-024-06648-z

**Published:** 2024-07-12

**Authors:** Mark A. Smith, Samantha P. Armas, Jacob D. Camp, Hannah N. Carlson

**Affiliations:** Department of Psychology and Program in Neuroscience, Davidson College, 209 Ridge Road, PO Box 5000, Davidson, NC 28035 USA

**Keywords:** Addiction, Animal model, Choice, Concurrent schedule, Estrous cycle, Progressive ratio, Sex differences, Social behavior

## Abstract

**Rationale:**

Preclinical studies report that drug use and social contact mutually influence the reinforcing effects of one another. Most of these studies have used same-sex dyads exclusively, and the role of factors related to biological sex and hormonal fluctuations are not well understood.

**Objectives:**

The purpose of this study was to examine the reinforcing effects of cocaine and social contact with an opposite-sex partner in male and female rats, and how these effects are modulated by ovarian hormones.

**Methods:**

Male and female rats were trained in a nonexclusive choice procedure in which cocaine and social contact with an opposite-sex partner were simultaneously available on concurrent progressive ratio schedules of reinforcement. To examine the effects of ovarian hormones related to estrous cycling, Experiment 1 used naturally cycling, gonadally intact females, whereas Experiment 2 used ovariectomized females, and estrus was artificially induced with exogenous hormones.

**Results:**

In both experiments, cocaine and social contact functioned as robust reinforcers, and there were no significant effects of biological sex or estrus status of the females. The positive reinforcing effects of both cocaine and social contact increased as a function of cocaine dose, indicating that contingent cocaine administration increases the reinforcing effects of social contact.

**Conclusions:**

These data suggest that cocaine use among opposite-sex partners may enhance factors that contribute to social bonding.

**Supplementary Information:**

The online version contains supplementary material available at 10.1007/s00213-024-06648-z.

## Introduction

Drug use is influenced by social contact with others. Epidemiological studies consistently report that one of the most reliable predictors of a person’s drug use is the amount and frequency of drug use by other individuals in that person’s social environment (e.g., friends, family members: Chassin et al. 1996; Sieving et al. 2000; Biederman et al. 2000; Bahr et al. 2005; Schinke et al. 2008; Ramirez et al. 2012; Schuler et al. 2019). These data are supported by both human and nonhuman laboratory studies, which have consistently revealed a causal role for social contact in drug use (see reviews by Bardo et al. 2013; Strickland and Smith 2014; Pelloux et al. 2019). A recurring finding of laboratory animal studies is that drug use increases if a social partner is simultaneously using drugs or using drugs at a high rate, and drug use decreases if a social partner is not using drugs or using drugs at a low rate (Smith 2012; Peitz et al. 2013; Smith et al. 2014; Robinson et al. 2016, 2017).

The recent introduction of novel operant conditioning chambers that permit a laboratory rat to intravenously self-administer drugs in immediate physical proximity to another rat has advanced our understanding of how drug use and social contact function as reinforcers. For instance, several studies have revealed that rats will maintain a state of “voluntary abstinence” from drugs (e.g., cocaine, heroin, methamphetamine) when given a mutually exclusive choice between drugs and social contact with a sex-matched partner (Venniro et al. 2018, 2019, 2021, 2022). These effects are sensitive to behavioral economic manipulations such as the dose of the drug available and the effort requirement (e.g., FR value on a fixed ratio schedule: Venniro et al. 2021; Chow et al. 2022; Marcus et al. 2022).

The effects of drug use and social contact are bidirectional, with each mutually influencing the positive reinforcing effects of the other. For instance, noncontingent administration of cocaine, d-amphetamine, and WIN-35,428 increases the positive reinforcing effects of social contact as measured on a progressive ratio (PR) schedule of reinforcement (Sharp and Smith 2021). Similarly, response-contingent social contact increases the positive reinforcing effects of cocaine (Smith et al. 2021), and response-contingent cocaine increases the positive reinforcing effects of social contact (Smith et al. 2023a). Rats also prefer to self-administer cocaine in close proximity to another rat self-administering cocaine (Smith and Pitts 2014) and prefer a rat with a shared history of cocaine exposure over another rat without a shared history (Smith et al. 2015). In a free-operant procedure of nonexclusive choice in which responding maintained by cocaine and social contact operated independently of one another, social contact served as a robust economic substitute for cocaine (Smith et al. 2023b).

The effects of social contact on drug self-administration are generally similar between males and females (cf., Smith 2012; Robinson et al. 2017), suggesting social learning processes relevant to drug use are conserved between males and females. Almost all previous studies have used same-sex dyads exclusively, and the question remains whether social contact with an opposite-sex partner influences drug intake in the same manner as described above for a same-sex partner. In laboratory animals, social preference for an opposite-sex partner is determined in large part by ovarian hormones, with both males and females exhibiting greater social interaction with an opposite-sex partner when the female is in a state of behavioral estrus (i.e., heat: Merkx 1983; López et al. 1999, 2007).

Recently, Chow et al. reported that operant responding maintained by an opposite-sex partner relative to a same-sex partner was greater in males than females, and this effect was not influenced by the estrous cycle (Chow et al. 2024). Drug self-administration was not examined in that study, and comparisons between cocaine and opposite-sex social contact were not determined. The purpose of the present study was to compare the reinforcing effects of cocaine and opposite-sex social contact in male and female rats in a free-operant procedure of nonexclusive choice. In this procedure, both cocaine and opposite-sex social contact were simultaneously available on concurrent PR schedules of reinforcement that operate independently of one another (i.e., the subject is free to allocate its responses between the two response alternatives without penalty). A second purpose of this study was to examine the role of the estrous cycle and behavioral estrus on the reinforcing effects of cocaine and opposite-sex contact. To this end, Experiment 1 used intact male and female rats, and responding was examined across the estrous cycle of females; Experiment 2 used intact male and ovariectomized female rats, and estrus was artificially induced in females by experimenter-delivered injections of estradiol and progesterone.

## Method

**Fig. 1:**
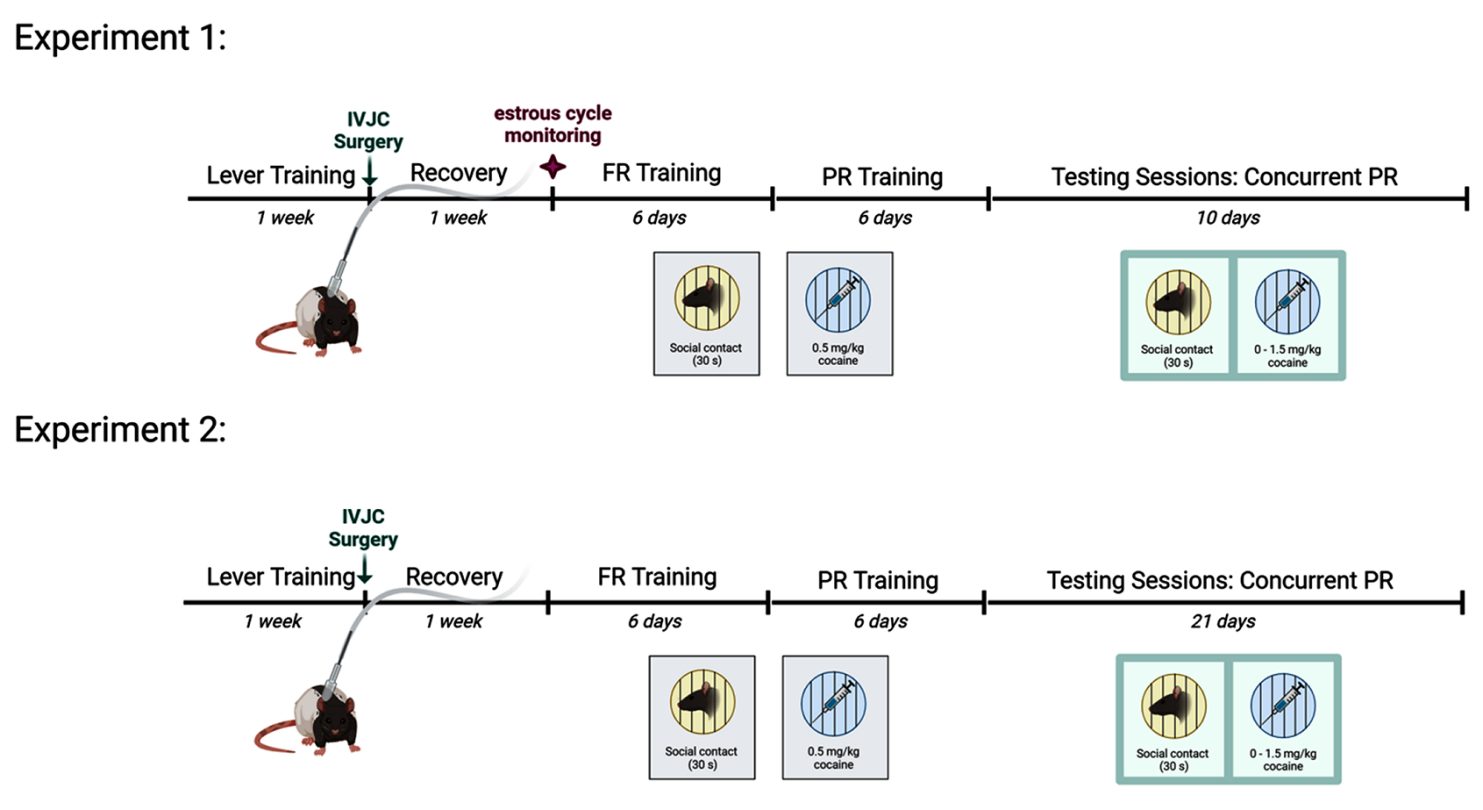
Timeline of events for Experiments 1 and 2. Created with BioRender.com

A timeline of both experiments is shown in Fig. 1.

### Experiment 1

#### Animals

Male and female Long-Evans rats were obtained from Charles River Laboratories (Raleigh, NC, USA) at 7 weeks of age. Rats were housed individually in a temperature- and humidity-controlled vivarium on a 12-hr light/dark cycle. Except for the brief period of lever press training (see below), food was continuously available in the home cage. Enrichment items (gnaw sticks, housing tubes, additional nesting materials) were added to the cage twice per week, and water was continuously available throughout the study.

#### Lever press training

One week after arrival, rats were lightly food restricted to 90% of their free-feeding body weight and trained to press a response lever using food reinforcement (45 mg grain pellets) on a fixed ratio (FR1) schedule of reinforcement in operant conditioning chambers from Med Associates, Inc (St. Albans, VT, USA). Training continued until rats received 40 reinforcers in at least two consecutive sessions. These training sessions were conducted in operant conditioning chambers different from those later used for drug/social self-administration testing.

#### Surgery

One week after lever-press training, rats were deeply anesthetized with inhaled isoflurane and surgically implanted with intravenous catheters into the right jugular vein. Ketoprofen was given immediately after surgery and the following morning as a post-operative analgesic. Ticarcillin was given intravenously for 7 consecutive days to prevent infection, and catheters were flushed daily with heparinized saline to maintain patency for the duration of the study.

#### Equipment

All testing was conducted in operant conditioning chambers from Med Associates, Inc. The chambers were located in a different room and had a different configuration from those used for lever-press training. These chambers contained a houselight, two response levers, two stimulus lights (one stimulus light located directly above each response lever), a syringe pump located immediately outside the chamber with a tethering system for intravenous drug self-administration, and an attached social compartment separated from the main chamber by a guillotine door that could be raised to allow social contact. A metal screen was affixed to the opening between the main chamber and the social compartment that permitted visual, auditory, olfactory, and limited tactile contact between two rats, but that prevented each rat from traversing from one compartment to the other (see description and photographs in Venniro and Shaham 2020). All operant conditioning chambers were housed in larger sound-attenuating chambers, and white noise was continuously present in the testing room to mask extraneous noise.

#### Estrous cycle monitoring

Beginning one week after surgery, the estrous cycle was monitored daily in all females, including females used as social partners. Each day, vaginal cells were collected via lavage 30–60 min before each test session. Cytology was performed using light microscopy (x 100 magnification) and categorized into one of four estrous phases (metestrus, diestrus, proestrus, and estrus) as described in previous studies (Marcondes et al. 2002; Hubscher et al. 2005; Goldman et al. 2007). Please see Schmidt et al. (2021) for representative images of vaginal cytology depicting all four phases of the estrous cycle for normally cycling female rats from our laboratory.

#### Behavioral training

Approximately one week after surgery, male and female rats were introduced to the testing chambers, and responding was reinforced with either (1) 0.3 mg/kg cocaine or (2) 30-s access to an age-matched opposite sex-partner on a fixed ratio (FR1) schedule of reinforcement). Each session began with illumination of the houselight and noncontingent delivery of the reinforcer available during that session (0.3 mg/kg cocaine or opening of the guillotine door for 30-s access to a social partner). After 30 s, a single lever was inserted into the cage and the stimulus light above the lever was illuminated. A single response on the lever turned off the light above the response lever, retracted the response lever, and activated either the syringe pump (cocaine reinforcer) or the guillotine door (social reinforcer). After 30 s, conditions reset, the lever was reinserted into the chamber, the stimulus light was illuminated, and the same reinforcer was again available on the FR1 schedule. Each session lasted 60 min and no limit was placed on the maximum number of reinforcers that could be earned. Training continued in this manner for six sessions, with cocaine and social reinforcement available during alternating sessions (three training sessions each). Throughout training, one lever was designated the social lever (always the lever closest to the guillotine door) and one lever was designated the cocaine lever (always the lever furthest from the guillotine door).

After six sessions, contingencies were changed, and responding was then maintained on a progressive ratio (PR) schedule for an additional six sessions. In these sessions, the number of responses required to obtain a reinforcer increased through the following progression: 1, 3, 6, 9, 12, 17, 24, 32, 42, 56, 73, 95, 124, 161, 208, and 268 (for complete algorithm, see Suto et al. 2002). Sessions terminated automatically once 60 min elapsed in which no reinforcers were delivered. Otherwise, all conditions were identical to those used on the FR schedule. Training continued in this manner for six sessions, with cocaine and social reinforcement available during alternating sessions.

#### Behavioral testing

Behavioral testing commenced after six training sessions on the PR schedule. During testing, each session began with noncontingent delivery of both reinforcers, both levers were inserted into the chambers, and both reinforcers (cocaine and social contact) were available on concurrent, PR schedules of reinforcement. During these sessions, the dose of cocaine available was increased to 0.5 mg/kg/infusion. Each PR schedule functioned independently of the other, and subjects were free to allocate their responses between the two levers throughout the session without penalty. Each reinforced response retracted the lever associated with that response, turned off the stimulus light above that response lever, and delivered the relevant reinforcer; however, it did not have any effect on the other response lever, stimulus light, or reinforcer. After 30 s, the lever was reinserted into the cage, the stimulus was illuminated, and the reinforcer was again available on the PR schedule of reinforcement. Sessions terminated automatically once 60 min elapsed in which no reinforcers were delivered. Testing continued in this manner for 10 sessions, ensuring that at least two full estrous cycles (typical estrous cycle duration: 4–5 days) were completed prior to study termination. All rats were checked for catheter patency at the end of testing with an infusion of ketamine through the catheter. Rats that lost catheter patency were removed from the study, and their data were not included.

#### Data analysis

Breakpoints were operationally defined as the number of reinforcers obtained and served as the primary dependent measure. Our intention was to compare breakpoints across metestrus/diestrus, proestrus, and estrus; however, estrus was not represented in all rats, and proestrus was represented in only a small minority of rats. Because behavioral estrus (i.e., the period of sexual receptivity) overlaps the phases of proestrus and estrus, and because vaginal cytology revealed that all rats were transitioning between proestus and estrus during at least one session, the phases of proestrus, estrus, and the transitional phase between proestrus and estrus were collapsed. Consequently, the factor of estrous phase contained two levels: metestrus/diestrus vs. proestrus/estrus. All data were subsequently analyzed via a 2 × 2, repeated-measures ANOVA, with reinforcer (cocaine vs. social contact) and estrous phase serving as factors. The effect of biological sex was also examined for each reinforcing stimulus using two-way, mixed-factor ANOVA, with sex serving as the between-subjects factor and estrous phase serving as the within-subject factor. Within-session patterns of responding maintained by cocaine and opposite-sex social contact were compared by visual analysis of cumulative records, supplemented by waffle plots and distribution plots.

### Experiment 2

#### Animals

Male and female Long-Evans rats were obtained from Charles River Laboratories (Raleigh, NC, USA) at 7 weeks of age. Male rats arrived intact, but female rats were ovariectomized by the vender at 6 weeks of age. All other characteristics and conditions were identical to those described in Experiment 1.

#### Lever press training

One week after arrival, rats were lightly food restricted to 90% of their free-feeding body weight and trained to press a response lever as described in Experiment 1.

#### Surgery

One week after lever-press training, rats were anesthetized with inhaled isoflurane and surgically implanted with intravenous catheters as described in Experiment 1.

#### Equipment

All testing was conducted in the same operant conditioning chambers from Med Associates, Inc. as described in Experiment 1.

#### Behavioral training

Behavioral training was identical to that described in Experiment 1, including the training dose of 0.3 mg/kg/infusion cocaine.

#### Behavioral testing

Experimental parameters during testing sessions were similar to those described in Experiment 1, with both reinforcers available on the PR schedule of reinforcement.

Testing lasted three weeks, with sessions conducted 5 days/week. Each week, the dose of cocaine was changed, and three doses of cocaine were tested: 0.0 mg/kg (saline), 0.5 mg/kg, and 1.5 mg/kg. No hormones were administered on Mondays, Tuesdays, or Wednesdays. No hormones were administered prior to sessions on Thursdays, but data obtained during Thursday sessions were used as the nonhormonal control data for data analytic purposes. Artificial estrus was induced by administering a bolus dose of estradiol (0.005 mg, sc) to females immediately after Thursday’s test session, followed by a bolus dose of progesterone (0.125 mg, sc) approximately 20 h later, 60 min before Friday’s test session. This and similar protocols reliably induce behavioral estrus and a state of sexual receptivity in ovariectomized female rats (Witcher and Freeman 1985; Saito 1987; Bivens and Olster 1997; Herath et al. 2001). Data collected in Friday’s test session were used to determine the effects of artificial estrus on breakpoints maintained by cocaine and social contact with an opposite-sex partner. All rats were checked for catheter patency at the end of testing with an infusion of ketamine through the catheter. Rats that lost catheter patency were removed from the study and their data were not analyzed.

### Data analysis

Similar to Experiment 1, breakpoints were operationally defined as the number of reinforcers obtained and served as the primary dependent measure. Data were initially analyzed via a three-way, repeated-measures ANOVA, with dose of cocaine, reinforcing stimulus, and artificial estrous status all serving as within-subject factors. The effects of biological sex were also examined for each reinforcing stimulus using three-way, mixed-factor ANOVA, with sex serving as the between subjects factor, and dose of cocaine and artificial estrus status serving as within-subject factors. Within-session patterns of responding maintained by cocaine and opposite-sex social contact were compared by visual analysis of cumulative records, supplemented by waffle plots and distribution plots.

## Results

Training data for Experiments 1 and 2 are shown in Supplemental Fig. 1.

### Experiment 1

**Fig. 2:**
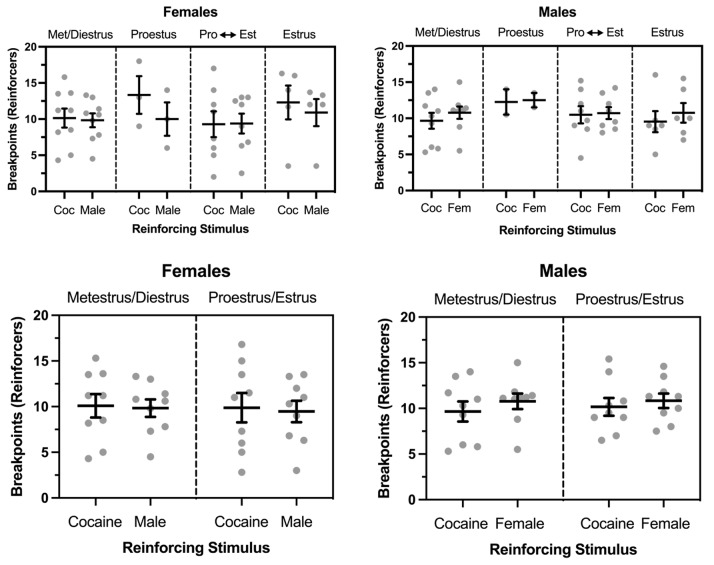
Upper Panels: Breakpoints maintained by cocaine and opposite-sex social contact in female (left: *n* = 9) and male (right: *n* = 9) rats under a concurrent PR-PR schedule of reinforcement. Data were collected when females (either responder females or female partners) were in either (1) metestrus or diestrus (met/diestrus), (2) proestrus, (3) during the transition between proestrus and estrus (Pro <--> Est), and (4) estrus. Lower Panels: Data from various stages of the female’s estrous cycle when collapsed into metestrus/diestrus or proestrus/estrus. Horizontal bars represent mean (± SEM). Gray dots represent data from individual subjects

Figure 2 depicts breakpoints maintained by a male social partner and cocaine (0.5 mg/kg) in females across all phases of the estrous cycle. As noted above (see Results), the phases of proestus and estrus were not represented in all rats, and only a visual analysis of these data were conducted. Generally, breakpoints were similar across estrous phases and between the two reinforcers. Figure 1 also depicts breakpoints maintained by a female social partner and cocaine in males across all phases of the partner’s estrous cycle. Again, the phases of proestrus and estrus were not represented in all rats, and only a visual analysis of the data were conducted. Breakpoints were generally similar between the two reinforcers across all phases of the partner’s estrous cycle.

Figure 2 also compares breakpoints maintained by a male social partner and cocaine in females in metestrus/diestrus vs. proestus/estrus. Breakpoints maintained by both reinforcers averaged approximately 10 reinforcers/session regardless of estrous phase, and no significant main effects or interactions were observed. Figure 2 also compares breakpoints maintained by a female social partner and cocaine in males when the social partner was in metestrus/diestrus vs. proestus/estrus. Breakpoints maintained by both reinforcers were similar to those observed in females regardless of the partner’s phase of the estrous cycle, and no significant main effects or interactions were observed.

**Fig. 3:**
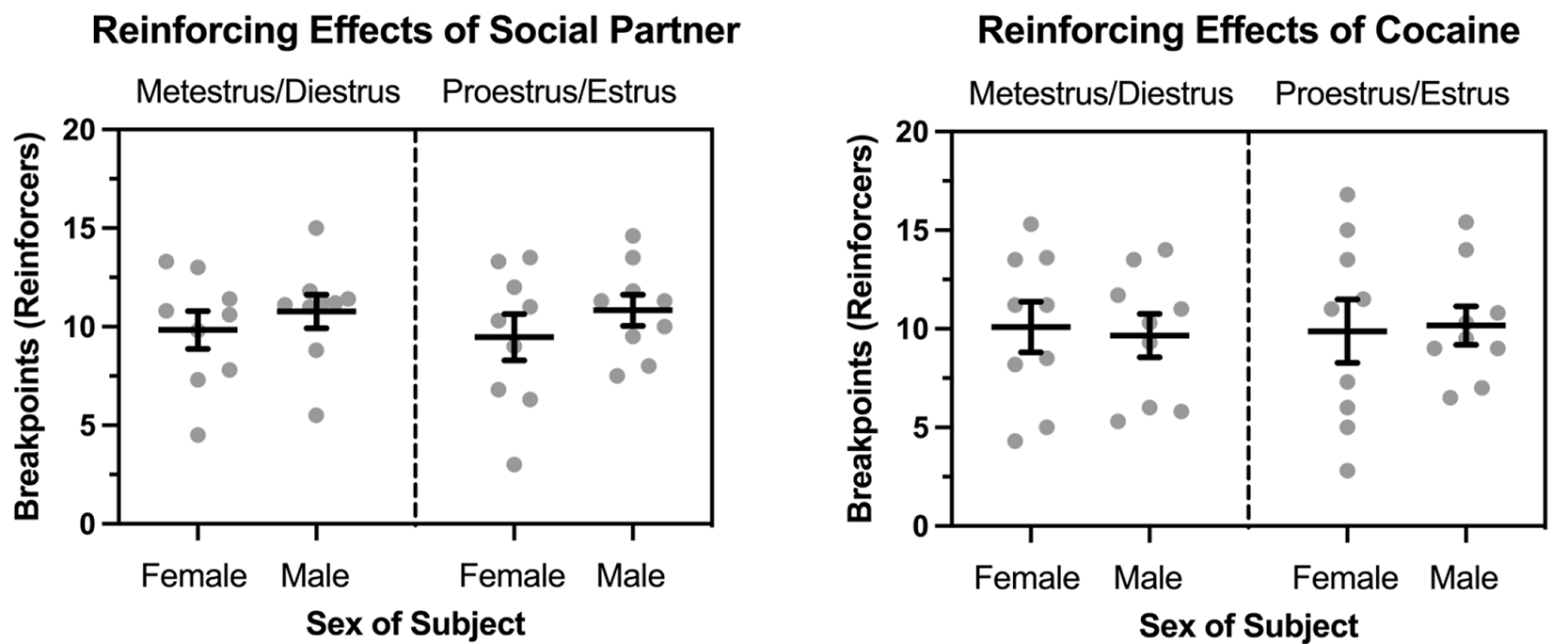
Breakpoints maintained by cocaine and opposite-sex social contact in female (left: *n* = 9) and male (right: *n* = 9) rats from Fig. 2. Data are shown separately for breakpoints maintained by cocaine and opposite-sex social contact to emphasize biological sex comparisons. Horizontal bars represent mean (± SEM). Gray dots represent data from individual subjects

Figure 3 depicts comparisons between males and females during metestrus/diestrus and proestrus/estrus for each reinforcer. Breakpoints were similar across sex and estrous cycle stages for both sexes and both reinforcers. No main effects or interactions involving biological sex were observed.

**Fig. 4:**
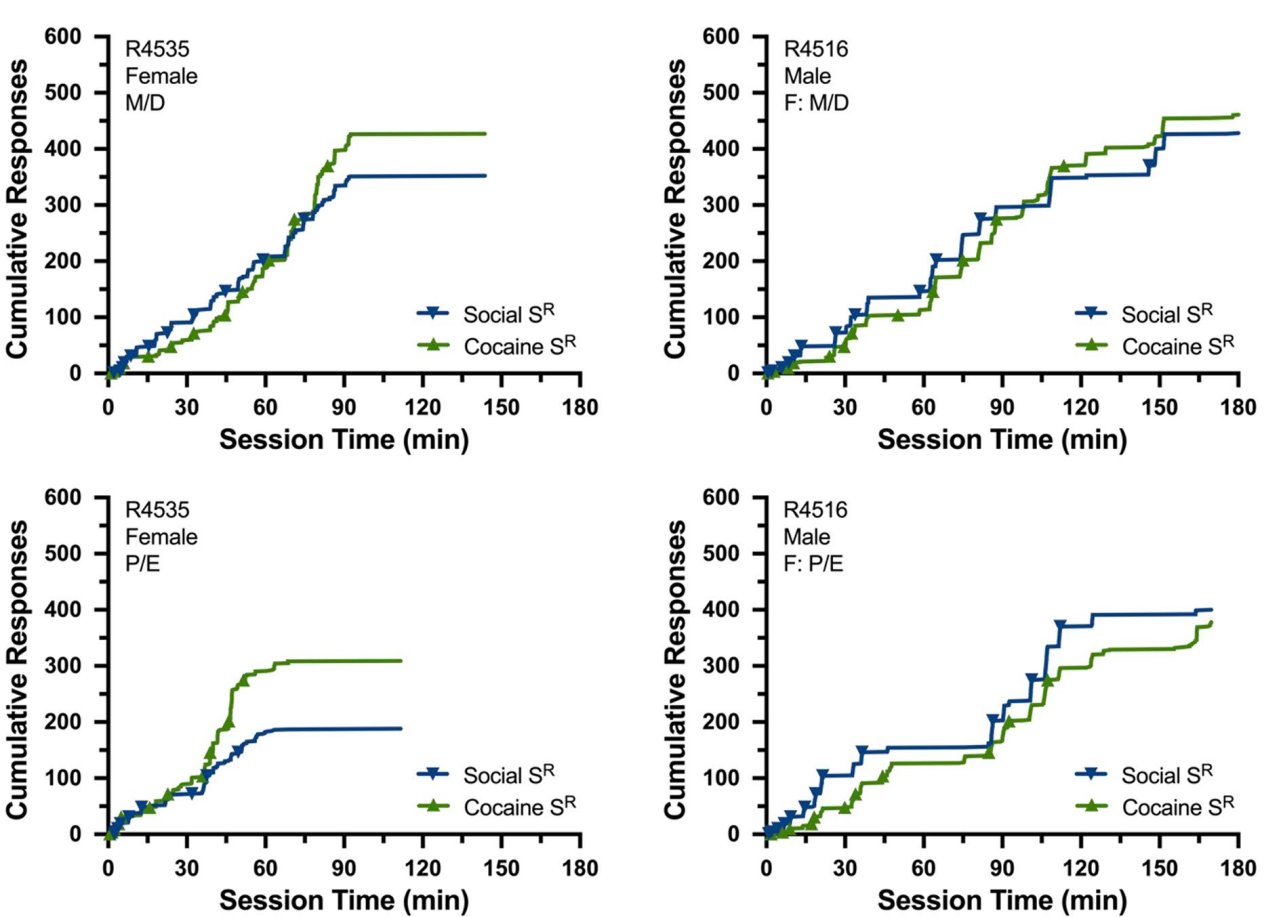
Cumulative records from the female (R4535: left) and male (R4516: right) rat most consistently representative of the group mean from Experiment 1 when the female (either responder female or female partner) was in metestrus/diestrus (top) or proestrus/estrus (bottom). Left axis depicts cumulative number of responses; bottom axis reflects time (min) in sessions. Cumulative records depict responding maintained by the social (blue) and cocaine (green) reinforcers. Reinforcer delivery noted by triangles

Temporal patterns of responding maintained by cocaine and opposite-sex social contact were examined via visual analysis of cumulative records, supplemented by waffle plots and distribution plots. Responding maintained by both reinforcers temporally overlapped for all subjects under all conditions. In other words, responding maintained by both reinforcers started at similar points in time, ratio runs occurred during similar intervals of time, post-reinforcement pauses occurred during similar intervals of time, and breakpoints were reached at similar points in time. Functionally, this pattern of responding meant that reinforcers generally alternated with one another throughout the session, including early in the session when ratio values were low and reinforcers were frequent, as well as well as later in the session when ratio values were high and reinforcers were infrequent. Furthermore, this general pattern was observed even when breakpoints differed between the two reinforcers. Figure 4 depicts cumulative recordings from the female (R4535) and male (R4516) rat most consistently representative of the group mean under metestrus/diestrus and proestrus/estrus. Supplemental Figs. 2 and 3 depict waffle plots for all individual subjects and distribution plots averaged across all subjects, respectively.

### Experiment 2

**Fig. 5:**
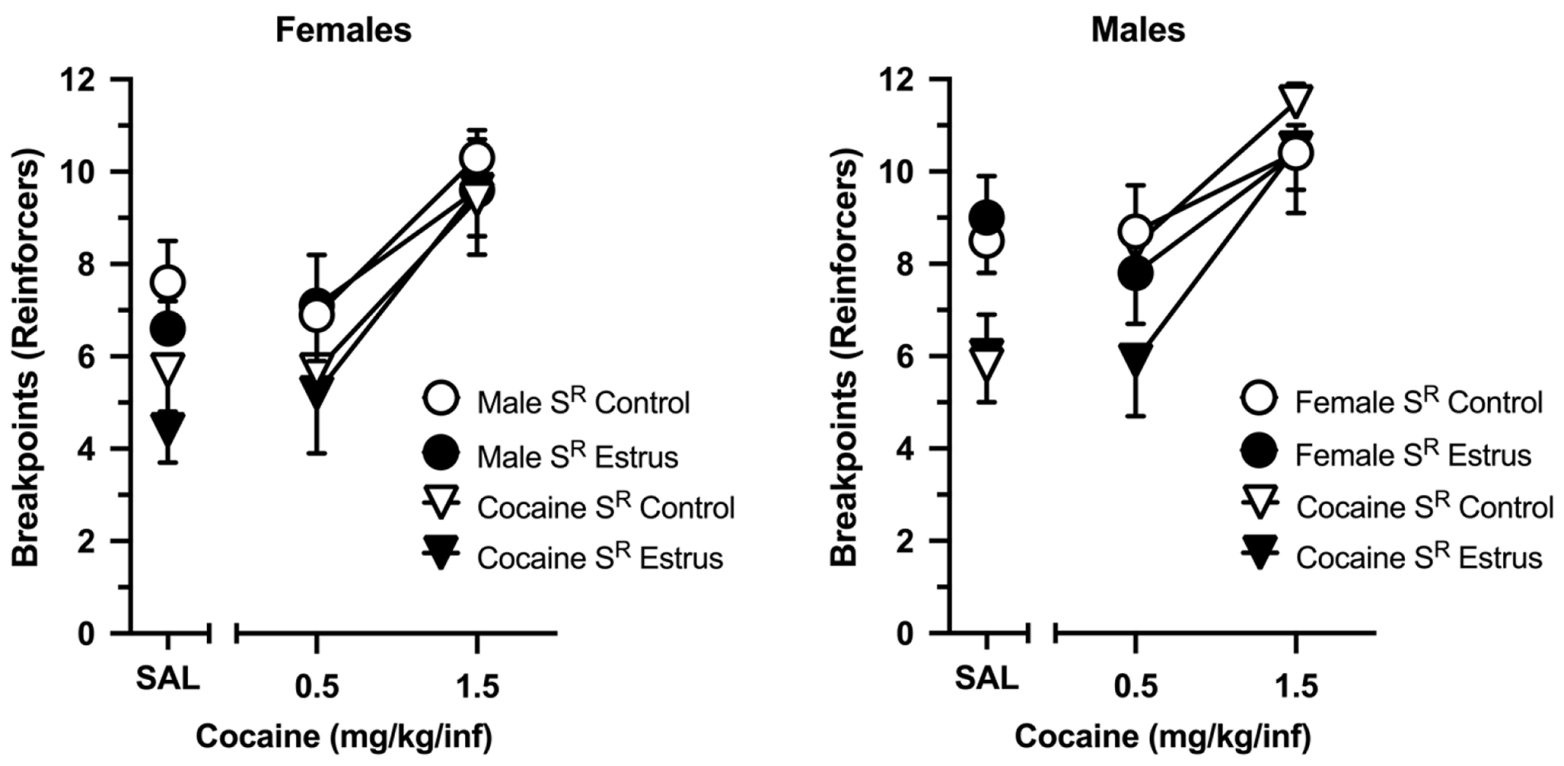
Breakpoints maintained by cocaine (triangles) and opposite-sex social contact (circles) in ovariectomized female (left: *n* = 9) and intact male (left: *n* = 10) rats under a concurrent PR-PR schedule of reinforcement. Left axis depicts breakpoints expressed as number of reinforcers obtained; bottom axis depicts doses of cocaine (or saline) in mg/kg/infusion. Data were collected under control (i.e., non-hormone: open symbols) conditions and under conditions in which estrus was artificially induced by exogenous hormone administration (filled symbols) in females. Data reflect the mean (± SEM)

Figure 5 depicts breakpoints maintained by cocaine and a social partner under control conditions and during artificial estrus. In females, breakpoints maintained by both cocaine and a male partner increased as a function of the dose of cocaine available (main effect of dose: *F* (2, 16) = 9.431, *p* = .002), and this effect was independent of estrus. Breakpoints maintained by a social partner were greater than those maintained by cocaine (main effect of stimulus: *F* (1, 8) = 10.766, *p* = .011), and this effect was consistent across all cocaine doses and independent of estrus. There were no main effects or interactions involving artificial estrus.

Similar effects were observed in males. Breakpoints maintained by both cocaine and a female partner increased as a function of the dose of cocaine available (main effect of dose: *F* (2, 18) = 6.624, *p* = .007), and this effect was independent of the estrus status of the female partner. Breakpoints maintained by social contact were greater than breakpoints maintained by cocaine, but this effect depended on the dose of cocaine (stimulus x dose interaction: *F* (2, 18) = 5.384, *p* = .015). Breakpoints maintained by the female partner were greater when the alternative was saline (*p* = .016), but differences diminished as the dose of cocaine increased. There were no main effects or interactions involving the estrus status of the female partner.

**Fig. 6:**
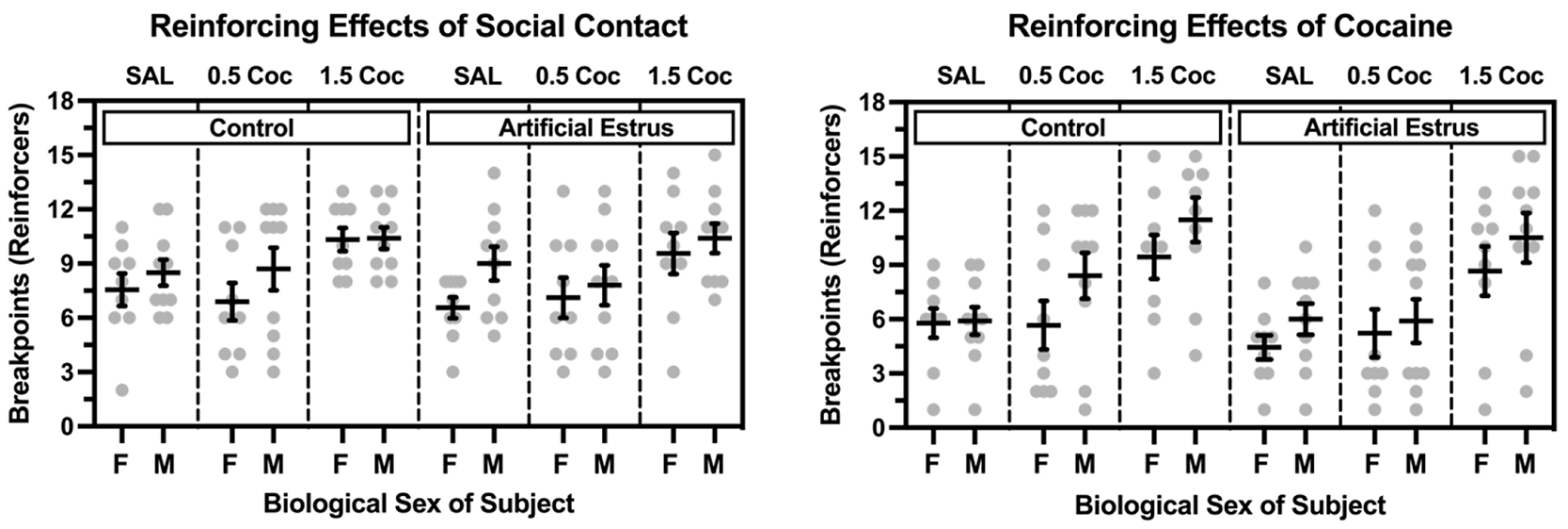
Breakpoints maintained by opposite-sex social contact (left) and cocaine (right) in ovariectomized female (*n* = 9) and intact male (*n* = 10) rats under a concurrent PR-PR schedule of reinforcement. Data are shown when responding on the cocaine-designated lever was reinforced with saline (SAL), 0.5 mg/kg/infusion cocaine (0.5 Coc), and 1.5 mg/kg/infusion cocaine (1.5 Coc), and when data were collected under control (i.e., non-hormone) conditions and under conditions in which estrus was artificially induced by exogenous hormone administration in females. Horizontal bars represent mean (± SEM). Gray dots represent data from individual subjects. This Fig. is created from the same dataset used for Fig. 5 and depicted to emphasize biological sex comparisons

Figure 6 depicts direct comparisons between males and females for each reinforcer. Breakpoints maintained by social contact increased in both sexes as a function of the dose of cocaine available (main effect of dose: *F* (2, 34) = 7.401, *p* = .002), but there were no main effects or interactions involving biological sex or estrus status of the female. Similarly, breakpoints maintained by cocaine increased in both sexes as a function of dose (main effect of dose: *F* (2, 34) = 19.948, *p* < .001), but there were no main effects or interactions involving biological sex or estrus status of the female.

**Fig. 7:**
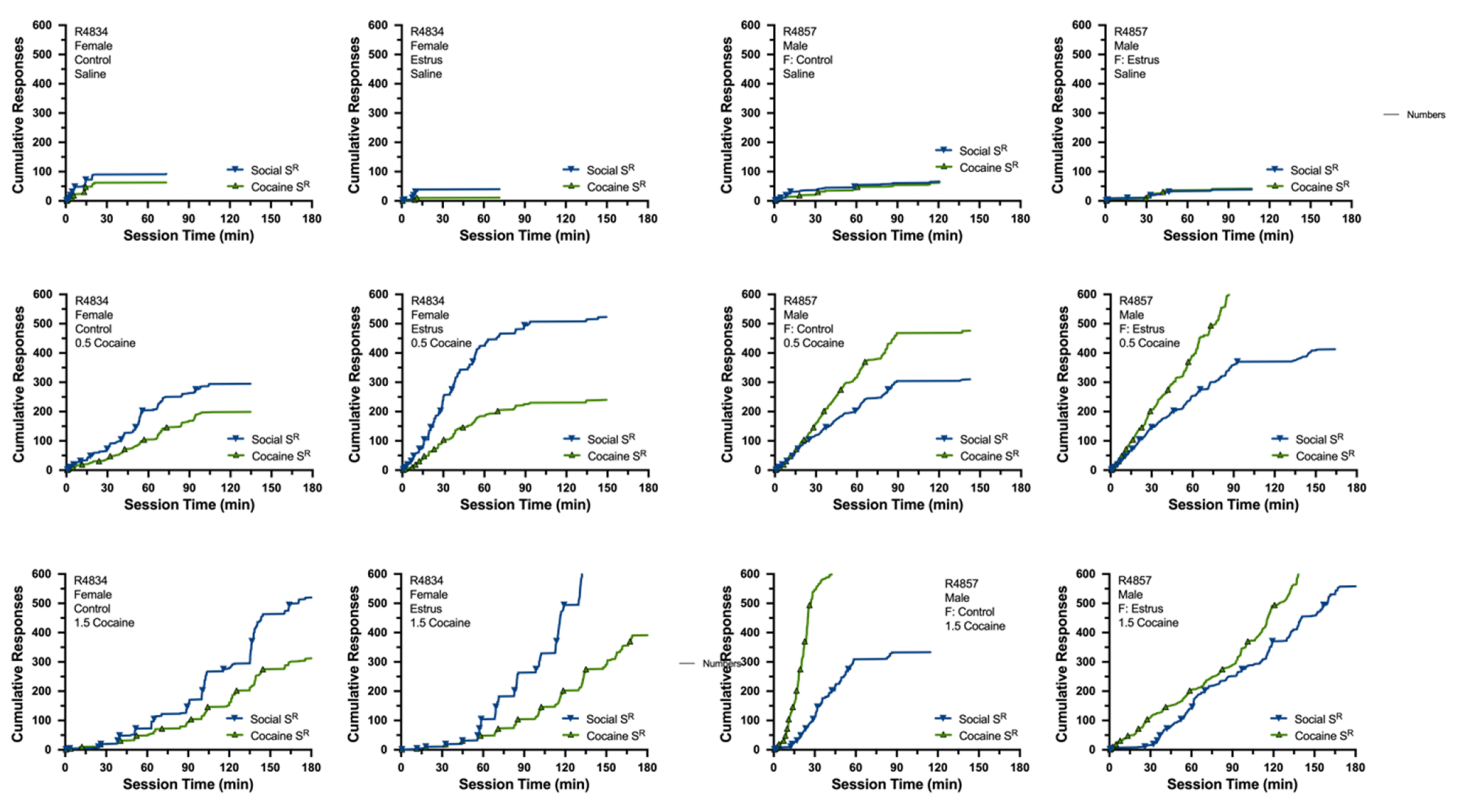
Cumulative records from the female (R4834: left columns) and male (R4857: right columns) rat most consistently representative of the group mean from Experiment 2. Data are shown when responding on the cocaine-assigned lever resulted in the delivery of 0.0 (saline: upper panels), 0.5 mg/kg (middle panels), or 1.5 mg/kg cocaine (lower panels). Data are also shown under control conditions (first and third column) and during artificially induced estrus (second and fourth columns). Left axis depicts cumulative number of responses; bottom axis reflects time (min) in sessions). Cumulative records depict responding maintained by the social (blue) and cocaine (green) reinforcers. Reinforcer delivery noted by triangles. All graphs truncated at 600 responses

Temporal patterns of responding maintained by cocaine and opposite-sex social contact were very similar to those described for Experiment 1. Specifically, responding maintained by both reinforcers started at similar points in time, ratio runs occurred during similar intervals of time, post-reinforcement pauses occurred during similar intervals of time, breakpoints were reached at similar points in time, and reinforcers generally alternated across the session. Figure 7 depicts cumulative recordings from the female (R4834) and male (R4857) rat most consistently representative of the group mean at each dose of cocaine under control and artificial estrus conditions. Supplemental Figs. 4 and 5 depict waffle plots for all individual subjects and distribution plots averaged across all subjects, respectively.

## Discussion

We previously reported that both cocaine and social contact with a same-sex social partner function as robust positive reinforcers in a free-operant, concurrent, FR-FR schedule of reinforcement (Smith et al. 2023b). The present study extends those findings to a concurrent PR-PR schedule of reinforcement and to opposite-sex social contact. These data are in contrast to those reported in discrete-trial choice procedures in which choices are limited to either drug administration or social contact on a single trial (Venniro et al. 2018, 2019, 2021, 2022). Those studies have consistently revealed that rats will choose one reinforcer to the exclusion of the other, depending on factors such as the dose of the drug available, reinforcer delay, and response requirement (Venniro et al. 2021; Chow et al. 2022; Marcus et al. 2022).

Preclinical studies frequently (e.g., Quigley et al. 2021; Towers et al. 2023; Bender and Torregrossa 2023) but not always (e.g., Griffin et al. 2007; Datta et al. 2017; Lacy et al. 2018; Engi et al. 2021) report females self-administer more cocaine than males on PR schedules of reinforcement. Similarly, a recent study reported that demand for opposite-sex social contact is greater than same-sex social contact in male rats (Chow et al. 2024). No sex differences were observed in breakpoints maintained by cocaine or opposite-sex social contact in either experiment in the present investigation. The most notable difference between the present study and previous studies was that both reinforcers were concurrently available in the two experiments described here, suggesting that sex differences depend on concurrent availability of alternative but non-mutually exclusive reinforcers. Additionally, one difference between the present study and Chow et al. (2024) was that rats in the latter study had experience with both same- and opposite-sex reinforcers throughout training, which may have influenced the relative reinforcing efficacy of social contact in general or opposite-sex social contact specifically.

Motivational measures of cocaine self-administration vary across the estrous cycle, with measures of both cocaine intake and cocaine seeking greater during estrus than other phases of the estrous cycle (Roberts et al. 1989; Kippin et al. 2005; Feltenstein and See 2007). We previously reported that breakpoints maintained by cocaine are greater during estrus than other phases under conditions very similar to those described here. Specifically, we reported breakpoints were greater during estrus than metestrus/diestrus and proestrus in female rats self-administering cocaine when an intact male rat was continuously present and self-administering cocaine in an adjacent compartment separated only be a wire screen (Lacy et al. 2016). Taken collectively, these data suggest that concurrent and contingent access to an opposite-sex partner, but not concurrent and noncontingent access to an opposite-sex partner, eliminates the effects of estrous cycling on cocaine intake; however, additional control groups are needed to confirm this possibility.

Both male and female rats engage with opposite-sex partners more during proestus and estrus than other stages of the estrous cycle (Merkx 1983; López et al. 1999, 2007). Behavioral estrus takes place during late proestrus and early estrus, and is associated with sexual receptivity (i.e., heat; Hardy 1972). Consequently, we predicted that breakpoints maintained by an opposite-sex partner would be greatest during proestus and estrus in gonadally intact female rats and when estrus was artificially induced in ovariectomized female rats. Contrary to these expectations, the reinforcing effects of opposite-sex social contact did not vary across the estrous cycle and were not influenced by artificially induced estrus. These data are consistent with a recent study reporting that operant responding maintained by opposite-sex social contact did not vary across the estrous cycle on an FR schedule of reinforcement (Chow et al. 2024).

The cocaine dose was varied in Experiment 2 to determine how responding maintained by the two reinforcers were influenced by the dose of cocaine available on the concurrent schedule. As expected, a high dose of cocaine maintained greater breakpoints than a low dose of cocaine, and this effect was independent of biological sex and estrus. Notably, breakpoints maintained by opposite-sex social contact also increased as a function of the cocaine dose available. We previously reported that experimenter-delivered (i.e., noncontingent) cocaine increases breakpoints maintained by same-sex social contact on a PR schedule (Sharp and Smith 2021), and that a history of response-contingent cocaine and social contact on an FR schedule later increases breakpoints maintained by social contact on a PR schedule (Smith et al. 2023a). The present data extend previous findings by showing that concurrent access to response-contingent cocaine enhances the reinforcing effects of opposite-sex social contact.

Dopamine regulates opposite-sex-directed sexual behavior in male (Beny-Shefer et al. 2017) and female (Micevych and Meisel 2017) mammals, and mediates social reward and reinforcement across species. For instance, dopamine increases measures of social play and approach in rodents (Trainor 2011; Manduca et al. 2016). Dopamine depletion in the prefrontal cortex decreases social interaction in rats (e.g., Fernandez Espejo 2003) and dopamine blockade in the lateral septum reduces social play in mice (Bredewold et al. 2018). In monogamous prairie voles, increases in dopamine in the nucleus accumbens facilitate pair bonding (Aragona et al. 2006), whereas blockade of dopamine in the nucleus accumbens prevents the development of a partner preference (Liu and Wang 2003) and decreases time spent with a familiar partner over an unfamiliar partner (Aragona et al. 2003). Cocaine, while decreasing social play, increases social exploration behaviors (e.g., sniffing, grooming; (Achterberg et al. 2014). Consequently, dopamine-mediated increases in the reinforcing effects of opposite-sex social exploration were likely responsible for the increases in breakpoints maintained by social contact in the present study.

An analysis of cumulative records and waffle plots for individual subjects revealed that responding maintained by cocaine and opposite-sex social contact were generally synchronized. In other words, responding maintained by the two reinforcers started at similar times, ratio runs occurred during similar periods of time, pauses occurred during similar periods of time, breakpoints were reached at similar times, and reinforcers generally alternated throughout the session. Given that rats were free to respond (or not respond) on both response alternatives without penalty, these data suggest that rats prefer simultaneous exposure to the two stimuli. These data further suggest that models using free-operant, nonexclusive-choice procedures may offer advantages over discrete-trial, forced-choice procedures under some translationally relevant conditions.

Limitations of this study should be addressed in future studies. For instance, sexual receptivity was not confirmed following artificially induced estrus, and no sexual activity was permitted throughout the study. The latter point is relevant because a history of sexual activity increases the rewarding effects of opposite-sex social contact in male rats (Tenk et al. 2009). Additionally, the reinforcing effects of cocaine and opposite-sex social contact were not determined in isolation, requiring that conclusions regarding the role of concurrent access be made by cross-study comparisons.

The observation that cocaine self-administration dose-dependently increased the positive reinforcing effects of opposite-sex social contact may explain the concordance of cocaine use among some populations (e.g. intimate partners). Specifically, cocaine use within intimate relationships likely increases the incentive salience of the partner, and this effect may be exaggerated if both partners engage in cocaine use. The reinforcing-enhancing effects of cocaine may thus enhance factors that contribute to social bonding, serving to maintain intimate relationships in which cocaine use is a norm.

## Electronic supplementary material

Below is the link to the electronic supplementary material.

Supplementary Material 1

Supplementary Material 2

Supplementary Material 3

Supplementary Material 4

Supplementary Material 5

## References

[CR1] Achterberg EJM, Trezza V, Siviy SM et al (2014) Amphetamine and cocaine suppress social play behavior in rats through distinct mechanisms. Psychopharmacology 231:1503–1515. 10.1007/s00213-013-3272-9 24057815 10.1007/s00213-013-3272-9 PMC3962711

[CR2] Aragona BJ, Liu Y, Curtis JT et al (2003) A critical role for nucleus accumbens dopamine in partner-preference formation in male prairie voles. J Neurosci 23:3483–3490. 10.1523/JNEUROSCI.23-08-03483.2003 12716957 10.1523/JNEUROSCI.23-08-03483.2003 PMC6742315

[CR3] Aragona BJ, Liu Y, Yu YJ et al (2006) Nucleus accumbens dopamine differentially mediates the formation and maintenance of monogamous pair bonds. Nat Neurosci 9:133–139. 10.1038/nn1613 16327783 10.1038/nn1613

[CR4] Bahr SJ, Hoffmann JP, Yang X (2005) Parental and peer influences on the risk of adolescent drug use. J Prim Prev 26:529–551. 10.1007/s10935-005-0014-8 16228115 10.1007/s10935-005-0014-8

[CR5] Bardo MT, Neisewander JL, Kelly TH (2013) Individual differences and social influences on the neurobehavioral pharmacology of abused drugs. Pharmacol Rev 65:255–290. 10.1124/pr.111.005124 23343975 10.1124/pr.111.005124 PMC3565917

[CR6] Bender BN, Torregrossa MM (2023) Intermittent cocaine self-administration has sex-specific effects on addiction-like behaviors in rats. Neuropharmacology 230:109490. 10.1016/j.neuropharm.2023.109490 36889433 10.1016/j.neuropharm.2023.109490 PMC10040443

[CR7] Beny-Shefer Y, Zilkha N, Lavi-Avnon Y et al (2017) Nucleus accumbens dopamine signaling regulates sexual preference for females in male mice. Cell Rep 21:3079–3088. 10.1016/j.celrep.2017.11.062 29241537 10.1016/j.celrep.2017.11.062

[CR8] Biederman J, Faraone SV, Monuteaux MC, Feighner JA (2000) Patterns of alcohol and drug use in adolescents can be predicted by parental substance use disorders. Pediatrics 106:792–797. 10.1542/peds.106.4.792 11015524 10.1542/peds.106.4.792

[CR9] Bivens CLM, Olster DH (1997) Abnormal estrous cyclicity and behavioral hyporesponsiveness to ovarian hormones in genetically obese zucker female rats1. Endocrinology 138:143–148. 10.1210/endo.138.1.4849 8977397 10.1210/endo.138.1.4849

[CR10] Bredewold R, Nascimento NF, Ro GS et al (2018) Involvement of dopamine, but not norepinephrine, in the sex-specific regulation of juvenile socially rewarding behavior by vasopressin. Neuropsychopharmacology 43:2109–2117. 10.1038/s41386-018-0100-2 29875448 10.1038/s41386-018-0100-2 PMC6098123

[CR11] Chassin L, Curran PJ, Hussong AM, Colder CR (1996) The relation of parent alcoholism to adolescent substance use: a longitudinal follow-up study. J Abnorm Psychol 105:70–80. 10.1037//0021-843x.105.1.70 10.1037//0021-843x.105.1.70 8666713

[CR12] Chow JJ, Beacher NJ, Chabot JM et al (2022) Characterization of operant social interaction in rats: effects of access duration, effort, peer familiarity, housing conditions, and choice between social interaction vs. food or remifentanil. Psychopharmacology 239:2093–2108. 10.1007/s00213-022-06064-1 35230469 10.1007/s00213-022-06064-1 PMC10724845

[CR13] Chow JJ, Pitts KM, Schoenbaum A et al (2024) Different effects of peer sex on Operant Responding for Social Interaction and Striatal dopamine activity. J Neurosci 44. 10.1523/JNEUROSCI.1887-23.2024 10.1523/JNEUROSCI.1887-23.2024 PMC10919252 38346894

[CR14] Datta U, Martini M, Sun WL (2017) Sex differences in the Motivational Contrast between sucrose and Cocaine in rats. J Drug Des Res 4:1042 34622250 PMC8494449

[CR15] Engi SA, Beebe EJ, Ayvazian VM et al (2021) Cocaine-induced increases in motivation require 2-arachidonoylglycerol mobilization and CB1 receptor activation in the ventral tegmental area. Neuropharmacology 193:108625. 10.1016/j.neuropharm.2021.108625 34058192 10.1016/j.neuropharm.2021.108625 PMC8312311

[CR16] Feltenstein MW, See RE (2007) Plasma progesterone levels and cocaine-seeking in freely cycling female rats across the estrous cycle. Drug Alcohol Depend 89:183–189. 10.1016/j.drugalcdep.2006.12.017 17240083 10.1016/j.drugalcdep.2006.12.017 PMC2099261

[CR17] Fernandez Espejo E (2003) Prefrontocortical dopamine loss in rats delays long-term extinction of contextual conditioned fear, and reduces social interaction without affecting short-term social interaction memory. Neuropsychopharmacology 28:490–498. 10.1038/sj.npp.1300066 12629528 10.1038/sj.npp.1300066

[CR18] Goldman JM, Murr AS, Cooper RL (2007) The rodent estrous cycle: characterization of vaginal cytology and its utility in toxicological studies. Birth Defects Res B Dev Reprod Toxicol 80:84–97. 10.1002/bdrb.20106 17342777 10.1002/bdrb.20106

[CR19] Griffin WC 3rd, Randall PK, Middaugh LD (2007) Intravenous cocaine self-administration: individual differences in male and female C57BL/6J mice. Pharmacol Biochem Behav 87:267–279. 10.1016/j.pbb.2007.04.023 17561241 10.1016/j.pbb.2007.04.023 PMC2692891

[CR20] Hardy DF (1972) Sexual behavior in continuously cycling rats. Behaviour 41:288–297. 10.1163/156853972x00068 5063409 10.1163/156853972x00068

[CR21] Herath CB, Watanabe G, Katsuda S et al (2001) Exposure of neonatal female rats to p-tert-octylphenol disrupts afternoon surges of luteinizing hormone, follicle-stimulating hormone and prolactin secretion, and interferes with sexual receptive behavior in adulthood. Biol Reprod 64:1216–1224. 10.1095/biolreprod64.4.1216 11259270 10.1095/biolreprod64.4.1216

[CR22] Hubscher CH, Brooks DL, Johnson JR (2005) A quantitative method for assessing stages of the rat estrous cycle. Biotech Histochem 80:79–87. 10.1080/10520290500138422 16195173 10.1080/10520290500138422

[CR23] Kippin TE, Fuchs RA, Mehta RH et al (2005) Potentiation of cocaine-primed reinstatement of drug seeking in female rats during estrus. Psychopharmacology 182:245–252. 10.1007/s00213-005-0071-y 16001116 10.1007/s00213-005-0071-y

[CR25] Lacy RT, Strickland JC, Feinstein MA et al (2016) The effects of sex, estrous cycle, and social contact on cocaine and heroin self-administration in rats. Psychopharmacology 233:3201–3210. 10.1007/s00213-016-4368-9 27370020 10.1007/s00213-016-4368-9 PMC5259804

[CR24] Lacy RT, Schorsch HK, Austin BP (2018) Adolescent d-amphetamine exposure enhances the acquisition of cocaine self-administration in male and female rats. Exp Clin Psychopharmacol 26:18–28. 10.1037/pha0000164 29389167 10.1037/pha0000164

[CR26] Liu Y, Wang ZX (2003) Nucleus accumbens oxytocin and dopamine interact to regulate pair bond formation in female prairie voles. Neuroscience 121:537–544. 10.1016/s0306-4522(03)00555-4 14568015 10.1016/s0306-4522(03)00555-4

[CR27] López HH, Olster DH, Ettenberg A (1999) Sexual motivation in the male rat: the role of primary incentives and copulatory experience. Horm Behav 36:176–185. 10.1006/hbeh.1999.1535 10506541 10.1006/hbeh.1999.1535

[CR28] López HH, Wurzel G, Ragen B (2007) The effect of acute bupropion on sexual motivation and behavior in the female rat. Pharmacol Biochem Behav 87:369–379. 10.1016/j.pbb.2007.05.014 17586031 10.1016/j.pbb.2007.05.014

[CR29] Manduca A, Servadio M, Damsteegt R et al (2016) Dopaminergic neurotransmission in the Nucleus Accumbens modulates Social Play Behavior in rats. Neuropsychopharmacology 41:2215–2223. 10.1038/npp.2016.22 26860202 10.1038/npp.2016.22 PMC4946055

[CR30] Marcondes FK, Bianchi FJ, Tanno AP (2002) Determination of the estrous cycle phases of rats: some helpful considerations. Braz J Biol 62:609–614. 10.1590/s1519-69842002000400008 12659010 10.1590/s1519-69842002000400008

[CR31] Marcus MM, Negus SS, Banks ML (2022) Effects of environmental manipulations on cocaine-vs-social choice in male and female rats. Pharmacol Biochem Behav 220:173462. 10.1016/j.pbb.2022.173462 36084838 10.1016/j.pbb.2022.173462 PMC10020861

[CR32] Merkx J (1983) Sexual motivation of the male rat during the oestrous cycle of the female rat. Behav Brain Res 7:229–237. 10.1016/0166-4328(83)90193-6 6681976 10.1016/0166-4328(83)90193-6

[CR33] Micevych PE, Meisel RL (2017) Integrating neural circuits Controlling female sexual behavior. Front Syst Neurosci 11:42. 10.3389/fnsys.2017.00042 28642689 10.3389/fnsys.2017.00042 PMC5462959

[CR34] Peitz GW, Strickland JC, Pitts EG et al (2013) Peer influences on drug self-administration: an econometric analysis in socially housed rats. Behav Pharmacol 24:114–123. 10.1097/FBP.0b013e32835f1719 23412112 10.1097/FBP.0b013e32835f1719 PMC3749306

[CR35] Pelloux Y, Giorla E, Montanari C, Baunez C (2019) Social modulation of drug use and drug addiction. Neuropharmacology 159:107545. 10.1016/j.neuropharm.2019.02.027 30807753 10.1016/j.neuropharm.2019.02.027

[CR36] Quigley JA, Logsdon MK, Graham BC et al (2021) Activation of G protein-coupled estradiol receptor 1 in the dorsolateral striatum enhances motivation for cocaine and drug-induced reinstatement in female but not male rats. Biol Sex Differ 12:46. 10.1186/s13293-021-00389-w 34391470 10.1186/s13293-021-00389-w PMC8364009

[CR37] Ramirez R, Hinman A, Sterling S et al (2012) Peer influences on adolescent alcohol and other drug use outcomes. J Nurs Scholarsh 44:36–44. 10.1111/j.1547-5069.2011.01437.x 22339982 10.1111/j.1547-5069.2011.01437.x PMC3287367

[CR38] Roberts DC, Bennett SA, Vickers GJ (1989) The estrous cycle affects cocaine self-administration on a progressive ratio schedule in rats. Psychopharmacology 98:408–411. 10.1007/BF00451696 2501818 10.1007/BF00451696

[CR40] Robinson AM, Lacy RT, Strickland JC et al (2016) The effects of social contact on cocaine intake under extended-access conditions in male rats. Exp Clin Psychopharmacol 24:285–296. 10.1037/pha0000078 27454676 10.1037/pha0000078 PMC4965182

[CR39] Robinson AM, Fronk GE, Zhang H et al (2017) The effects of social contact on cocaine intake in female rats. Drug Alcohol Depend 177:48–53. 10.1016/j.drugalcdep.2017.03.027 28558271 10.1016/j.drugalcdep.2017.03.027 PMC5534368

[CR41] Saito TR (1987) Copulatory behavior of male rats paired with natural proestrous and hormone-treated ovariectomized females. Jikken Dobutsu 36:91–93. 10.1538/expanim1978.36.1_91 3816995 10.1538/expanim1978.36.1_91

[CR42] Schinke SP, Fang L, Cole KCA (2008) Substance use among early adolescent girls: risk and protective factors. J Adolesc Health 43:191–194. 10.1016/j.jadohealth.2007.12.014 18639794 10.1016/j.jadohealth.2007.12.014 PMC2517143

[CR43] Schmidt KT, Sharp JL, Ethridge SB et al (2021) The effects of strain and estrous cycle on heroin- and sugar-maintained responding in female rats. Behav Brain Res 409:113329. 10.1016/j.bbr.2021.113329 33933523 10.1016/j.bbr.2021.113329 PMC8137667

[CR44] Schuler MS, Tucker JS, Pedersen ER, D’Amico EJ (2019) Relative influence of perceived peer and family substance use on adolescent alcohol, cigarette, and marijuana use across middle and high school. Addict Behav 88:99–105. 10.1016/j.addbeh.2018.08.025 30173075 10.1016/j.addbeh.2018.08.025 PMC6314679

[CR45] Sharp JL, Smith MA (2021) The effects of drugs on Behavior maintained by Social Contact: role of monoamines in social reinforcement. Front Behav Neurosci 15:805139. 10.3389/fnbeh.2021.805139 35264935 10.3389/fnbeh.2021.805139 PMC8899311

[CR46] Sieving RE, Perry CL, Williams CL (2000) Do friendships change behaviors, or do behaviors change friendships? Examining paths of influence in young adolescents’ alcohol use. J Adolesc Health 26:27–35. 10.1016/s1054-139x(99)00056-7 10638715 10.1016/s1054-139x(99)00056-7

[CR47] Smith MA (2012) Peer influences on drug self-administration: social facilitation and social inhibition of cocaine intake in male rats. Psychopharmacology 224:81–90. 10.1007/s00213-012-2737-6 22588251 10.1007/s00213-012-2737-6 PMC3752977

[CR52] Smith MA, Pitts EG (2014) Social preference and drug self-administration: a preclinical model of social choice within peer groups. Drug Alcohol Depend 135:140–145. 10.1016/j.drugalcdep.2013.12.001 24374111 10.1016/j.drugalcdep.2013.12.001 PMC3931128

[CR51] Smith MA, Lacy RT, Strickland JC (2014) The effects of social learning on the acquisition of cocaine self-administration. Drug Alcohol Depend 141:1–8. 10.1016/j.drugalcdep.2014.04.025 24878249 10.1016/j.drugalcdep.2014.04.025 PMC4102004

[CR53] Smith MA, Strickland JC, Bills SE, Lacy RT (2015) The effects of a shared history of drug exposure on social choice. Behav Pharmacol 26:631–635. 10.1097/FBP.0000000000000139 25932718 10.1097/FBP.0000000000000139 PMC4556544

[CR49] Smith MA, Cha HS, Griffith AK, Sharp JL (2021) Social Contact reinforces Cocaine Self-Administration in Young Adult male rats: the role of social reinforcement in vulnerability to Drug Use. Front Behav Neurosci 15:771114. 10.3389/fnbeh.2021.771114 34776897 10.3389/fnbeh.2021.771114 PMC8588844

[CR48] Smith MA, Camp JD, Johansen AN, Strickland JC (2023a) Response-contingent cocaine increases the reinforcing effectiveness of social contact. Exp Clin Psychopharmacol. 10.1037/pha0000679 37707472 10.1037/pha0000679 PMC10937325

[CR50] Smith MA, Cha HSH, Sharp JL, Strickland JC (2023b) Demand and cross-price elasticity of cocaine and social contact in a free-operant procedure of nonexclusive choice. Pharmacol Biochem Behav 222:173511. 10.1016/j.pbb.2022.173511 36572113 10.1016/j.pbb.2022.173511 PMC9845135

[CR54] Strickland JC, Smith MA (2014) The effects of social contact on drug use: behavioral mechanisms controlling drug intake. Exp Clin Psychopharmacol 22:23–34. 10.1037/a0034669 24188170 10.1037/a0034669 PMC3926100

[CR55] Suto N, Austin JD, Tanabe LM et al (2002) Previous exposure to VTA amphetamine enhances cocaine self-administration under a progressive ratio schedule in a D1 dopamine receptor dependent manner. Neuropsychopharmacology 27:970–979. 10.1016/S0893-133X(02)00379-2 12464454 10.1016/S0893-133X(02)00379-2

[CR56] Tenk CM, Wilson H, Zhang Q et al (2009) Sexual reward in male rats: effects of sexual experience on conditioned place preferences associated with ejaculation and intromissions. Horm Behav 55:93–97. 10.1016/j.yhbeh.2008.08.012 18835271 10.1016/j.yhbeh.2008.08.012 PMC2659494

[CR57] Towers EB, Williams IL, Qillawala EI, Lynch WJ (2023) Role of nucleus accumbens dopamine 2 receptors in motivating cocaine use in male and female rats prior to and following the development of an addiction-like phenotype. Front Pharmacol 14:1237990. 10.3389/fphar.2023.1237990 37564182 10.3389/fphar.2023.1237990 PMC10411909

[CR58] Trainor BC (2011) Stress responses and the mesolimbic dopamine system: social contexts and sex differences. Horm Behav 60:457–469. 10.1016/j.yhbeh.2011.08.013 21907202 10.1016/j.yhbeh.2011.08.013 PMC3217312

[CR62] Venniro M, Shaham Y (2020) An operant social self-administration and choice model in rats. Nat Protoc 15:1542–1559. 10.1038/s41596-020-0296-6 32203485 10.1038/s41596-020-0296-6 PMC8409109

[CR63] Venniro M, Zhang M, Caprioli D et al (2018) Volitional social interaction prevents drug addiction in rat models. Nat Neurosci 21:1520–1529. 10.1038/s41593-018-0246-6 30323276 10.1038/s41593-018-0246-6 PMC7386559

[CR61] Venniro M, Russell TI, Zhang M, Shaham Y (2019) Operant social reward decreases incubation of Heroin craving in male and female rats. Biol Psychiatry 86:848–856. 10.1016/j.biopsych.2019.05.018 31326085 10.1016/j.biopsych.2019.05.018 PMC8383184

[CR60] Venniro M, Panlilio LV, Epstein DH, Shaham Y (2021) The protective effect of operant social reward on cocaine self-administration, choice, and relapse is dependent on delay and effort for the social reward. Neuropsychopharmacology 46:2350–2357. 10.1038/s41386-021-01148-6 34400784 10.1038/s41386-021-01148-6 PMC8580997

[CR59] Venniro M, Marino RAM, Chow JJ et al (2022) The protective effect of social reward on opioid and psychostimulant reward and relapse: Behavior, Pharmacology, and brain regions. J Neurosci 42:9298–9314. 10.1523/JNEUROSCI.0931-22.2022 36517252 10.1523/JNEUROSCI.0931-22.2022 PMC9794371

[CR64] Witcher JA, Freeman ME (1985) The proestrous surge of prolactin enhances sexual receptivity in the rat. Biol Reprod 32:834–839. 10.1095/biolreprod32.4.834 4039953 10.1095/biolreprod32.4.834

