# Supplementary material for: The positive reinforcing effects of cocaine and opposite-sex social contact: roles of biological sex and estrus
Source: Psychopharmacology (Berl). 2024 Jul 12;242(1):71–83. doi: 10.1007/s00213-024-06648-z (PMC11742770; doi:10.1007/s00213-024-06648-z)
Supplement: Supplementary file 1 — Supplementary Material 1 [file 213_2024_6648_MOESM1_ESM.docx]

**Supplemental Figure 1**


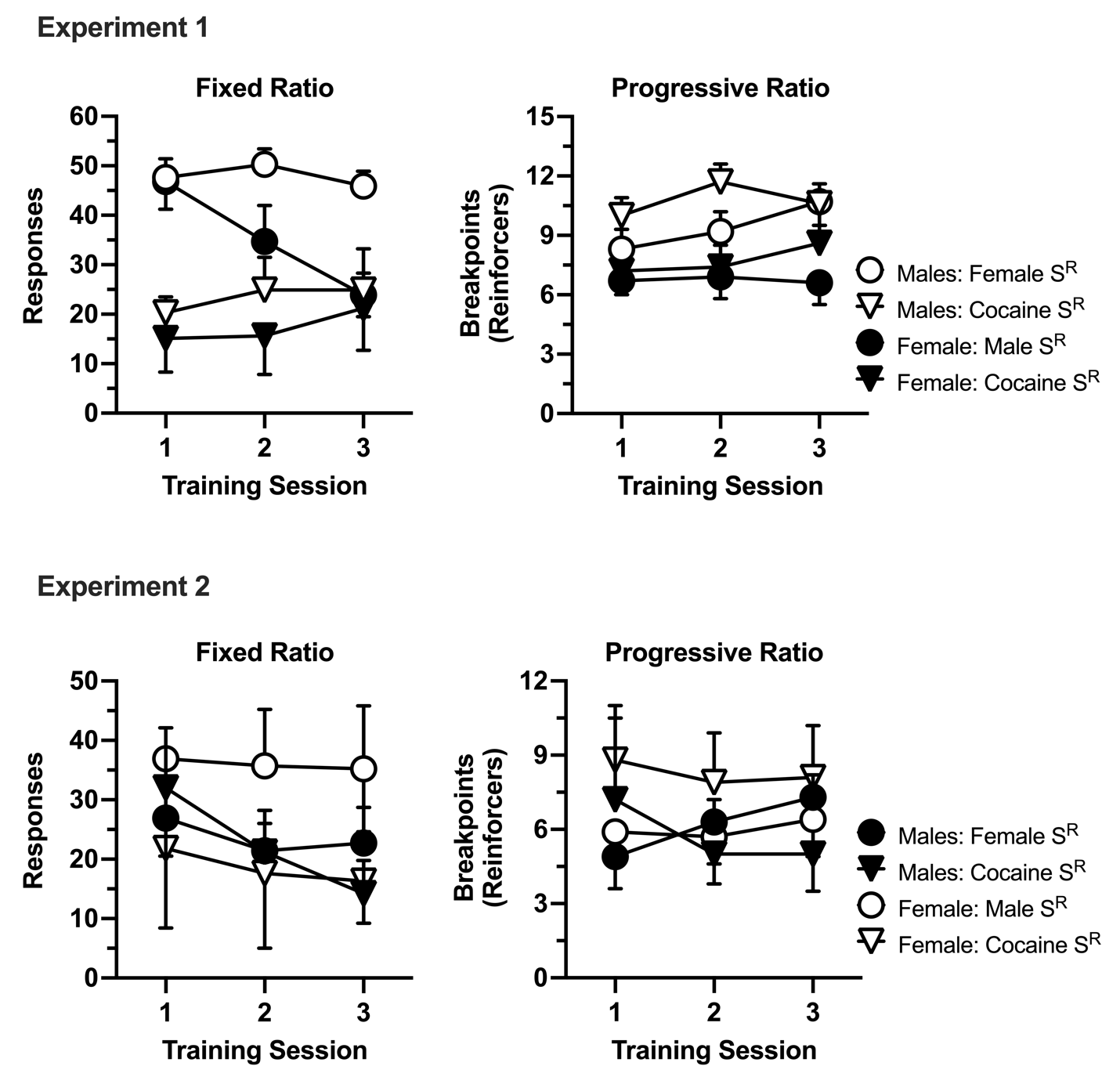


**Supplemental Figure 1.** Training data for Experiments 1 and 2. Training sessions consisted of 6 days of training on an FR1 schedule in which responding was reinforced by either 3.0 mg/kg/infusion cocaine (3 sessions) or 30-s access to an opposite sex partner (3 sessions), followed by 6 days of training on a PR schedule in which responding was reinforced by either 3.0 mg/kg/infusion cocaine (3 sessions) or 30-s access to an opposite sex partner (3 sessions). Significant effects were seen in Experiment 1 only. Fixed Ratio: main effect of stimulus (*F*[1, 16] = 17.811, *p* < .001), stimulus x day (*F* [2, 32] = 6.442, *p* = .004); Progressive Ratio: main effect of day (*F* [2, 32] = 10.176, *p* = .029; main effect of sex (*F* [1, 16] = 5.490, *p* = .032).
