# Supplementary material for: The positive reinforcing effects of cocaine and opposite-sex social contact: roles of biological sex and estrus
Source: Psychopharmacology (Berl). 2024 Jul 12;242(1):71–83. doi: 10.1007/s00213-024-06648-z (PMC11742770; doi:10.1007/s00213-024-06648-z)
Supplement: Supplementary file 3 — Supplementary Material 3 [file 213_2024_6648_MOESM3_ESM.docx]

**Supplemental Figure 3**

**Experiment 1: Females and Males**

**
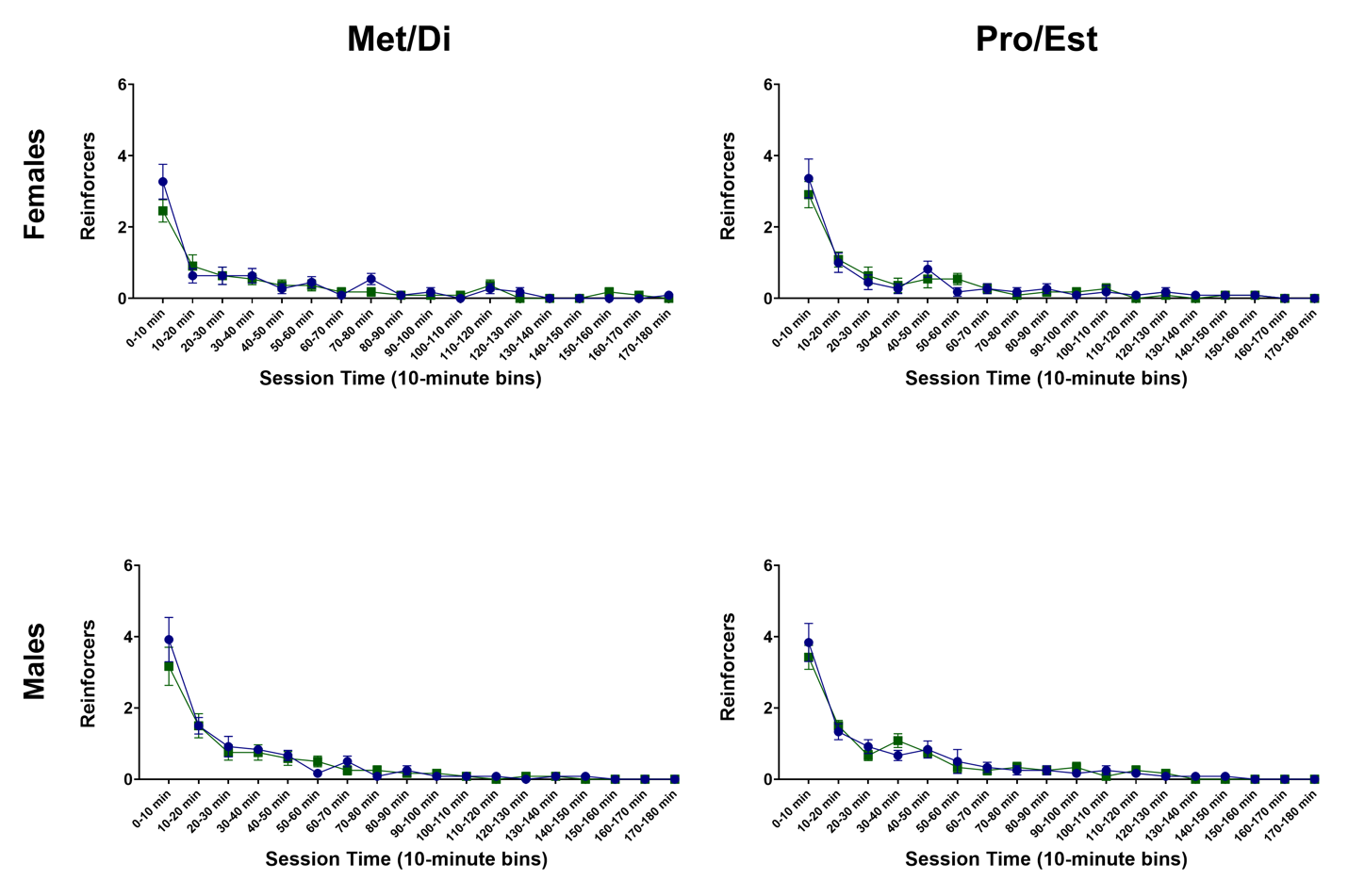
**

**Supplemental Figure 3.** Distribution plots of social (blue) and drug (green) reinforcers across session in Experiment 1. The y axis represents the number of reinforcers earned and the x axis denotes time across session in 10-minute increments (total session = 180 minutes). The same female (n = 11) and male (n = 12) rats are represented in each estrous condition (metestrus/diestrus vs. proestrus/estrus). Similar numbers of social and cocaine reinforcers were obtained in each bin, including early in the session when ratio values were low and reinforcers were frequent, and later in the session when ratio values were high and reinforcers were infrequent.
