# Supplementary material for: The positive reinforcing effects of cocaine and opposite-sex social contact: roles of biological sex and estrus
Source: Psychopharmacology (Berl). 2024 Jul 12;242(1):71–83. doi: 10.1007/s00213-024-06648-z (PMC11742770; doi:10.1007/s00213-024-06648-z)
Supplement: Supplementary file 4 — Supplementary Material 4 [file 213_2024_6648_MOESM4_ESM.docx]

**Supplemental Figure 4**

**Experiment 2: Females**

**
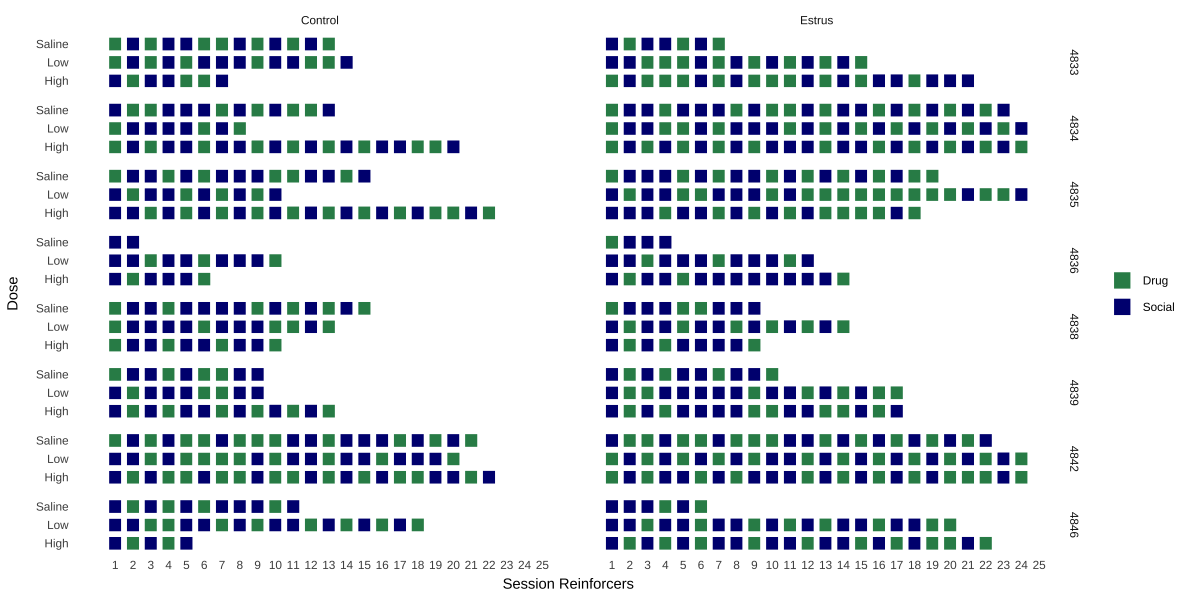
**

**Males: Males**


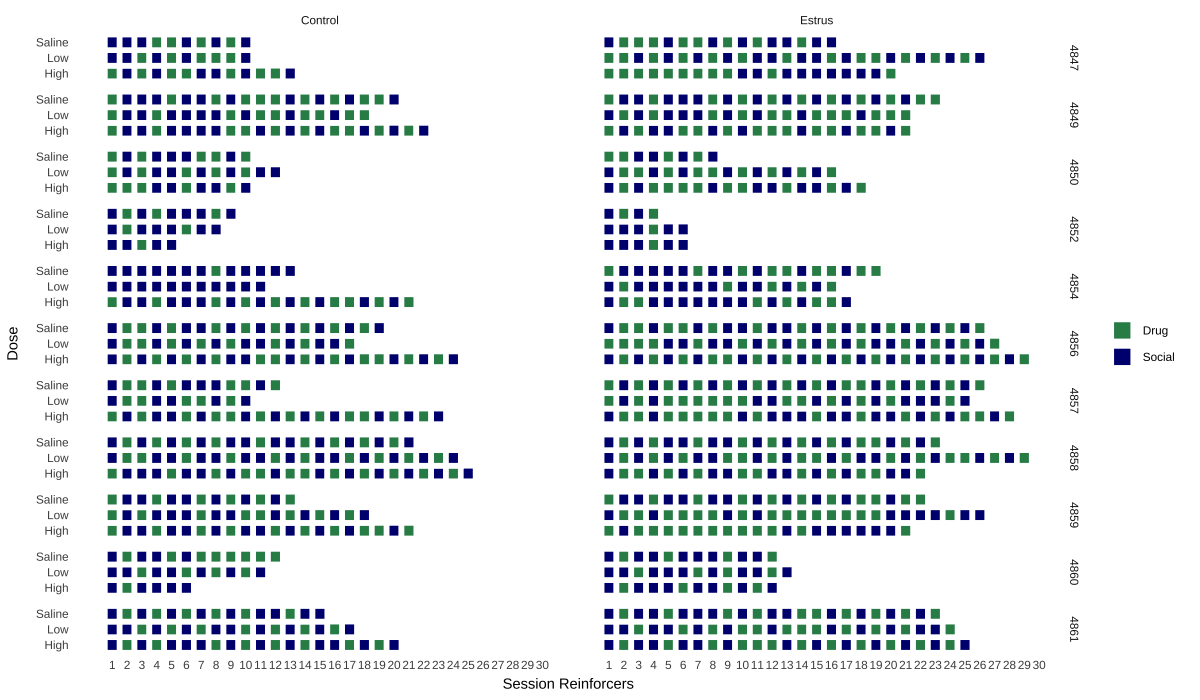


**Supplemental Figure 4.** Waffle plots depicting social (blue) and drug (green) reinforcers across session in order of procurement in Experiment 2. Subjects are presented individually, with a plot for each of six conditions (estrus x drug dose). Rows are arranged by individual and the dose of cocaine available during the session, and columns are arranged by the estrus phase of the female (responder or partner). Reinforcers generally alternated throughout the session for all subjects.
