# Supplementary material for: The positive reinforcing effects of cocaine and opposite-sex social contact: roles of biological sex and estrus
Source: Psychopharmacology (Berl). 2024 Jul 12;242(1):71–83. doi: 10.1007/s00213-024-06648-z (PMC11742770; doi:10.1007/s00213-024-06648-z)
Supplement: Supplementary file 5 — Supplementary Material 5 [file 213_2024_6648_MOESM5_ESM.docx]

**Supplemental Figure 5**

**Experiment 2: Females and Males**

**
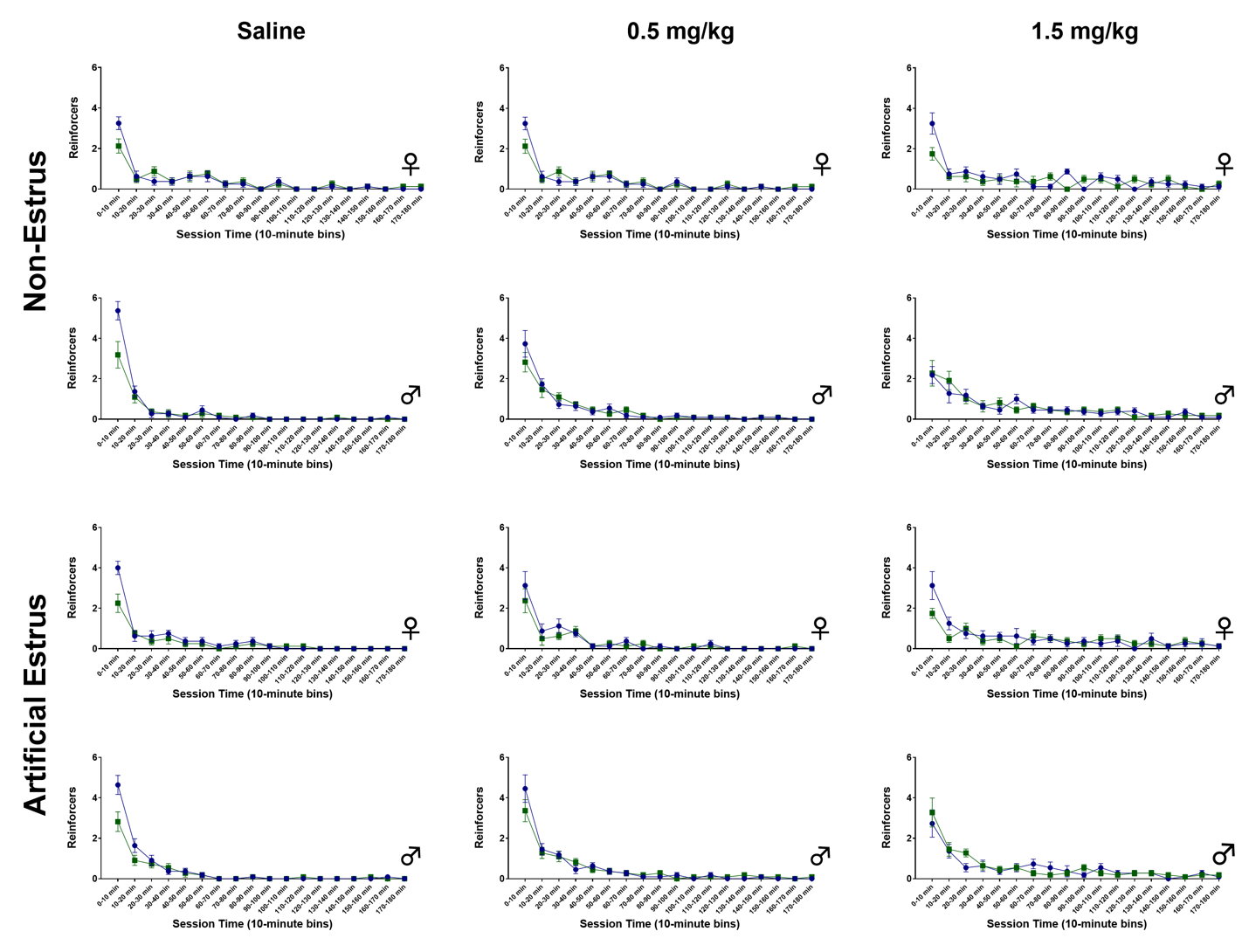
**

**Supplemental Figure 5.** Distribution plots of social (blue) and drug (green) reinforcers across session in Experiment 2. The y axis represents the number of reinforcers earned and the x axis denotes time across session in 10-minute increments (total session = 180 minutes). The same female (n = 9) and male (n = 11) rats are represented in each of six conditions (estrus X drug dose). Rows are arranged by sex of the responder and estrous status of the female (responder or partner). Columns are arranged by the dose of cocaine available during the session. Similar numbers of social and cocaine reinforcers were obtained in each bin, except under saline conditions in which greater number of social reinforcers were obtained during the first 10 min of the session.
